# Exploring racial and diagnostic differences in motor performance along the Alzheimer's disease continuum: Insights from the ARMADA study

**DOI:** 10.1002/alz70857_102194

**Published:** 2025-12-25

**Authors:** Kylie R. Kadey, Subhamoy Pal, Bruno Giordani, Aubryn H Samaroo, Emily H Ho, Richard C. Gershon, Sandra Weintraub, Vitali Ustsinovich, Amanda Cook Maher

**Affiliations:** ^1^ University of Michigan, Ann Arbor, MI, USA; ^2^ Michigan Alzheimer's Disease Research Center, Ann Arbor, MI, USA; ^3^ Massachusetts General Hospital, Boston, MA, USA; ^4^ Northwestern University Feinberg School of Medicine, Chicago, IL, USA; ^5^ Northwestern ADC Neuropathology Core, Northwestern University Feinberg School of Medicine, Chicago, IL, USA

## Abstract

**Background:**

Motor impairments are emerging predictors of amnestic mild cognitive impairment (aMCI) and dementia of the Alzheimer's type (DAT), with these groups demonstrating worse performance on metrics such as gait speed and endurance relative to cognitively normal (NC) controls (Windham et al., 2022). Racial disparities, including systemic inequities in healthcare access and chronic disease prevalence, contribute to worse motor performance in Black individuals (Blanco et al., 2012). This study examines NIH Toolbox Motor Battery (NIHTB‐MB) performance across racial and diagnostic groups (e.g., NC, aMCI, DAT), hypothesizing poorer motor performance in Black participants and linear motor decline across diagnostic categories.

**Method:**

The sample included 557 older adults ages 65‐99 (41.1% male) from the Assessing Reliable Measurement in Alzheimer's Disease and Cognitive Aging (ARMADA) study. Participants completed NIHTB‐MB measures of balance, gait speed, endurance, grip strength, and fine motor dexterity. After controlling for age and sex, racial differences between Black (*n* = 123) and White (*n* = 232) NC participants were assessed using multiple linear regression, while diagnostic group differences (NC: *n* = 355; aMCI: *n* = 137; DAT: *n* = 65) were examined using ANCOVA with post‐hoc comparisons.

**Result:**

Motor performance declined with age across all measures (*p* < .001). Black participants scored higher than White on Standing Balance, *t*(69)=‐2.91, *p* = .005, with no other racial differences in motor performance. Across diagnostic groups, individuals with DAT performed worse than NC on balance (*p* = .007), dominant/non‐dominant grip strength (*p* = .005/.05), dominant/non‐dominant dexterity (*p* = .003/< .001), and endurance (*p* < .001) measures. Participants with DAT also performed worse than those with aMCI on dominant/non‐dominant dexterity (*p* = .049/< .001), endurance (*p* = .002), and balance (*p* = .05). Gait speed did not differ across groups, and no motor differences were observed between NC and aMCI groups.

**Conclusion:**

Contrary to expectation, Black older adults exhibited better balance than White, though missing values were highest for this measure. Motor decline was primarily observed in individuals with DAT, who performed worse on fine motor dexterity, grip strength, endurance, and balance compared to NC and aMCI groups. These findings suggest that motor difficulty may serve as a marker of DAT, while NIHTB‐MB performance in aMCI remains largely preserved. Thus, more sensitive metrics, such as dual task conditions, may better characterize early motor changes in aMCI.

